# Supplementary material for: Assessment of established techniques to determine developmental and malignant potential of human pluripotent stem cells
Source: Nat Commun. 2018 May 15;9:1925. doi: 10.1038/s41467-018-04011-3 (PMC5954055; doi:10.1038/s41467-018-04011-3)
Supplement: Supplementary file 1 — Supplementary Information [file 41467_2018_4011_MOESM1_ESM.pdf]

SUPPLEMENTARY MATERIAL

TABLES, FIGURE LEGENDS and REFERENCES

Supplementary Table 1. Summary of the cell lines, culture conditions, teratoma production conditions used in this study

Lab	Cell Lines				Xenograft Tumor Production‡			
	Name (Culture and QC)*,◊	Other Name	Type	Ref#	PSC injected per mouse‡	Mouse Strain§	Sex	Period of tumor growth
Lab 1	H9 [a,e]	WA09	hESC	1	1-2 x 10^6	FOX CHASE SCID C.B-17/lcr scid/scidJcl (i)	Male	8-10 weeks
	KhES-1 [a,f]		hESC	2				
	201B7 [b,g]		hiPSC	3				
	TIG108-4F3 [b,h]		hiPSC	4				
Lab 2	H9 [c,e]	WA09	hESC	1	2 x 10^6	NOD.Cg-$Prkdc^{scid}Il2rg^{tm1Wjl}$/SzJ (ii)	Female	6-8 weeks
	HES3 *MIXL1*$^{GFP/w}$ [c,j]		hESC	5				
	MEL1 *INS*$^{GFP/w}$ [c,j]		hESC	6				
	iPSC RM3.5 [c,k]		hiPSC	7				
Lab 3	H9 [a,l]	WA09	hESC	1	6 x 10^6	SCID-Beige (iii)	Female	6-10 weeks
	H14 [a,l]	WA14	hESC	1				
	DF19-9-11T.H [a,l]		hiPSC	8				
	iPS(IMR90)-4 [a,l]		hiPSC	9				
Lab 4	H9 [d,m]	WA09	hESC	1	5 x 10^6	NOD.CB17-*Prkdc*^{scid}/ J (ii)	Female	14-16 weeks
	Shef3 [d,m]	Shef-3	hESC	10				
	Oxford-2 [d,m]	OXF2	hESC	11				
	NIBSC 5 [d,m]	NIBSC5	hiPSC	Unpub				

Notes:

*** Cell Line Culture Conditions**

a: DMEM/F12 + 20% KOSR, 5ng/mL bFGF, MEF feeders.
b: DMEM/F12 + 20% KOSR, 5ng/mL bFGF, SNL feeders.
c: DMEM/F-12 + 20% KOSR, 10ng/ml FGF2, MEF feeders.
d: mTeSR1, Matrigel

◊ Cell line STR and mycoplasma testing

e: Common stock from WiCELL, Banks were tested for Mycoplasma and STR analysis
f: Repeatedly tested for mycoplasma and STR, RIKEN BioResource Center confirmed negative for mycoplasma and its identity
g: Deposited to a cell bank in Japan (RIKEN BioResource Center). STR data was also reported in the reference of the cell line.
h: Tested for mycoplasma contamination. STR analysis has not been performed.
j: Cell lines were routinely tested for mycoplasma, MEL1, HES3 were purchased and validated by the vendor.
k: Cell line was routinely tested for mycoplasma, fingerprinted by SNP analysis.
l: Lines fingerprinted by STR, cell bank routinely tested for mycoplasma.
m: Lines fingerprinted by STR, cells tested for mycoplasma.

# See Supplementary References ‡

Xenograft Tumor Production

0.5 – 1 x 10^6 MEFs co-injected subcutaneously.

§ Origin of immuno-deficient mice

(i) CLEA, 1-2-7, Higashiyama, Meguro-ku, Tokyo 153-8533, JAPAN
(ii) Jackson Laboratories, 600 Main Street, Bar Harbor, ME USA 04609
(iii) Charles River Laboratories, 251 Ballardvale St, Wilmington, MA 01887, USA

Origin of Cell Lines

H9: WiCell Research Institute.

KhES-1: Institute for Frontier Medical Sciences; Kyoto University; Kyoto; Japan.

201B7: Center for iPS Cell Research and Application; Kyoto University; Kyoto; Japan.

TIG108-4F3: Center for iPS Cell Research and Application; Kyoto University; Kyoto; Japan.

HES3 *MIXL1*$^{GFP/w}$: Murdoch Childrens Research Institute

MEL1 *INS*$^{GFP/w}$: Murdoch Childrens Research Institute

iPSC RM3.5 : Murdoch Childrens Research Institute

H14: WiCell Research Institute

DF19-9-11T.H: WiCell Research Institute.

iPS(IMR90)-4: WiCell Research Institute.

Shef3: National Institute for Biological Standards and Control

Oxford-2: National Institute for Biological Standards and Control

NIBSC 5: National Institute for Biological Standards and Control

Supplementary Table 2. Marker gene list used for EB analysis.

Undifferentiated	Ectoderm	Mesoderm	Endoderm
TNFRSF8	*TH*	*PECAM1*	*FABP1*
TERT	*PAX3*	*CSF1R*	*ALB*
FOXD3	*MAP2*	*ITGB3*	*FOXA1*
MYC	*OTX2*	*VCAM1*	*HNF1A*
ALPL	*MAPT2*	*ANPEP*	*LRP2*
UTF1	*PAX7*	*IGFBP3*	*HNF1B*
POU5F1	*EN1*	*CDH5*	*SST*
NANOG	*NOG*	*SPI1*	*ISL1*
E2F1	*MNX1*	*THBD*	*DLX5*
PODXL	*CRABP2*	*ITGB2*	*SPARC*
GREM1	*PDGFRA*	*PTPRC*	*GATA6*
FGF2	*SNAI2*	*CD36*	*FABP2*
SHH	*SOX9*	*BMP2*	*HTATSF1*
DPPA3	*S100B*	*CD34*	*SLC2A2*
LIN28	*NEFL*	*CD14*	*GCG*

List of lineage markers and markers of undifferentiated PSC used for the analysis of the EB differentiation data

Supplementary Table 3. RNA-seq derived expression in teratomas of genes associated with PSC and/or yok sac.

Gene Name	Relative mean expression			Fold Change	P Value
	Pilot ◆	Total ●	Teratomas with undifferentiated/primitive cells■		
DNMT3B	2.2%	1.5%±0.2%	4.7%±0.8%	3.2	3.7E-06
DPPA4	3.1%	1.6%±0.2%	5.0%±0.9%	3.2	5.2E-06
GAL	0.2%	0.4%±0.1%	0.6%±0.2%	1.5	~0.2 (*N.S.*)
GDF3	4.4%	3.0%±0.2%	8.0%±1.4%	2.7	7.6E-06
LIN28A	2.9%	1.0%±0.3%	12.3%±1.8%	12.3	1.6E-10
NANOG	2.2%	1.3%±0.0%	7.5%±2.0%	5.9	2.0E-05
POU5F1	1.4%	0.4%±0.2%	4.0%±0.9%	10.2	1.6E-06
SALL4	1.2%	0.8%±0.1%	2.8%±0.4%	3.7	1.9E-07
TDGF1	2.2%	0.5%±0.1%	4.2%±1.0%	8.8	1.6E-06
ZFP42	5.6%	3.6%±0.7%	7.3%±1.2%	2.0	5.0E-03
**Average**	2.5%	1.4%±0.1%	5.6%±0.6%	4.1	3.4E-11

Ten pluripotency-associated markers were selected from an ISCI study of human PSC characteristics [12] as an indicator for residual undifferentiated cell presence. Gene expression is presented as the percentage of expression in pluripotent cells. Teratomas suspected to harbor residual undifferentiated pluripotent stem and/or yolk sac cells expressed higher levels of all 10 markers. (N.S. not significant). P-values were calculated using Student's t-test.

SUPPLEMENTARY REFERENCES

1. Thomson JA, *et al.* Embryonic stem cell lines derived from human blastocysts. *Science* **282**, 1145-1147 (1998).

2. Suemori H, Yasuchika K, Hasegawa K, Fujioka T, Tsuneyoshi N, Nakatsuji N. Efficient establishment of human embryonic stem cell lines and long-term maintenance with stable karyotype by enzymatic bulk passage. *Biochem Biophys Res Commun* **345**, 926-932 (2006).

3. Takahashi K, *et al.* Induction of pluripotent stem cells from adult human fibroblasts by defined factors. *Cell* **131**, 861-872 (2007).

4. Koyanagi-Aoi M, *et al.* Differentiation-defective phenotypes revealed by large-scale analyses of human pluripotent stem cells. *Proc Natl Acad Sci U S A* **110**, 20569-20574 (2013).

5. Davis RP, *et al.* Targeting a GFP reporter gene to the MIXL1 locus of human embryonic stem cells identifies human primitive streak-like cells and enables isolation of primitive hematopoietic precursors. *Blood* **111**, 1876-1884 (2008).

6. Micallef SJ, *et al.* INS(GFP/w) human embryonic stem cells facilitate isolation of in vitro derived insulin-producing cells. *Diabetologia* **55**, 694-706 (2012).

7. Kao T, *et al.* GAPTrap: A Simple Expression System for Pluripotent Stem Cells and Their Derivatives. *Stem Cell Reports* **7**, 518-526 (2016).

8. Yu J, *et al.* Induced pluripotent stem cell lines derived from human somatic cells. *Science* **318**, 1917-1920 (2007).

9. Yu J, *et al.* Human induced pluripotent stem cells free of vector and transgene sequences. *Science* **324**, 797-801 (2009).

10. Aflatoonian B, Ruban L, Shamsuddin S, Baker D, Andrews P, Moore H. Generation of Sheffield (Shef) human embryonic stem cell lines using a microdrop culture system. *In Vitro Cell Dev Biol Anim* **46**, 236-241 (2010).

11. Brook FA. Single-cell enzymatic dissociation of hESC lines OxF1-OxF4 and culture in feeder-free conditions. *Methods Mol Biol* **873**, 209-215 (2012).

12. Adewumi O, *et al.* Characterization of human embryonic stem cell lines by the International Stem Cell Initiative. *Nat Biotechnol* **25**, 803-816 (2007).

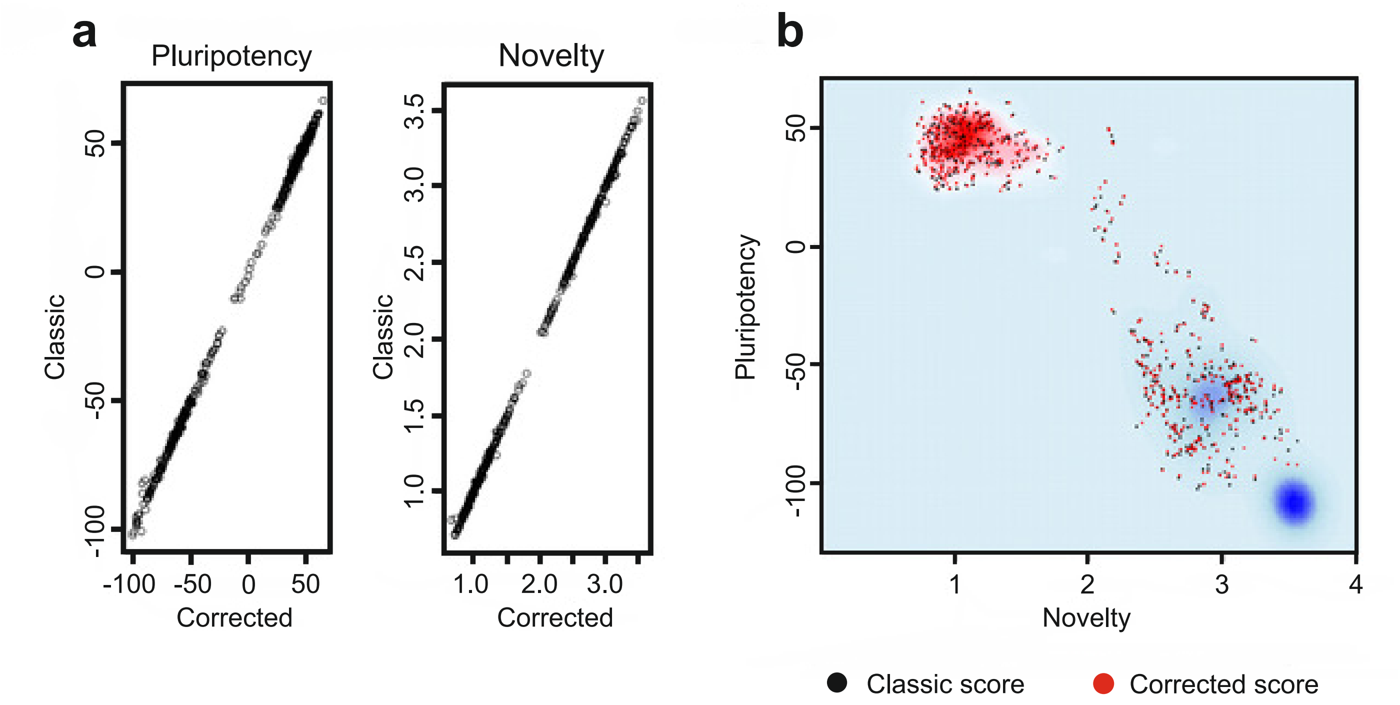

Supplementary Figure 1: Adapted PluriTest analysis. PluriTest algorithm was adapted to a new Illumina microarray platform. The modified algorithm was tested on the original training dataset to guarantee consistent results. **a** Plotting the "classic" vs. the "corrected" pluripotency and Novelty scores of the original training and test datasets demonstrates minimal changes to the scores of a few samples in the original dataset mainly on the differentiated category spectrum. **b** Plotting the same values within the pluripotency vs. novelty score plot also shows only a minimal shift between the classic (black dots) and the corrected values (red dots) in context. As a result of this correction, none of the training/test samples change position in regard to the Pluripotency and Novelty thresholds.

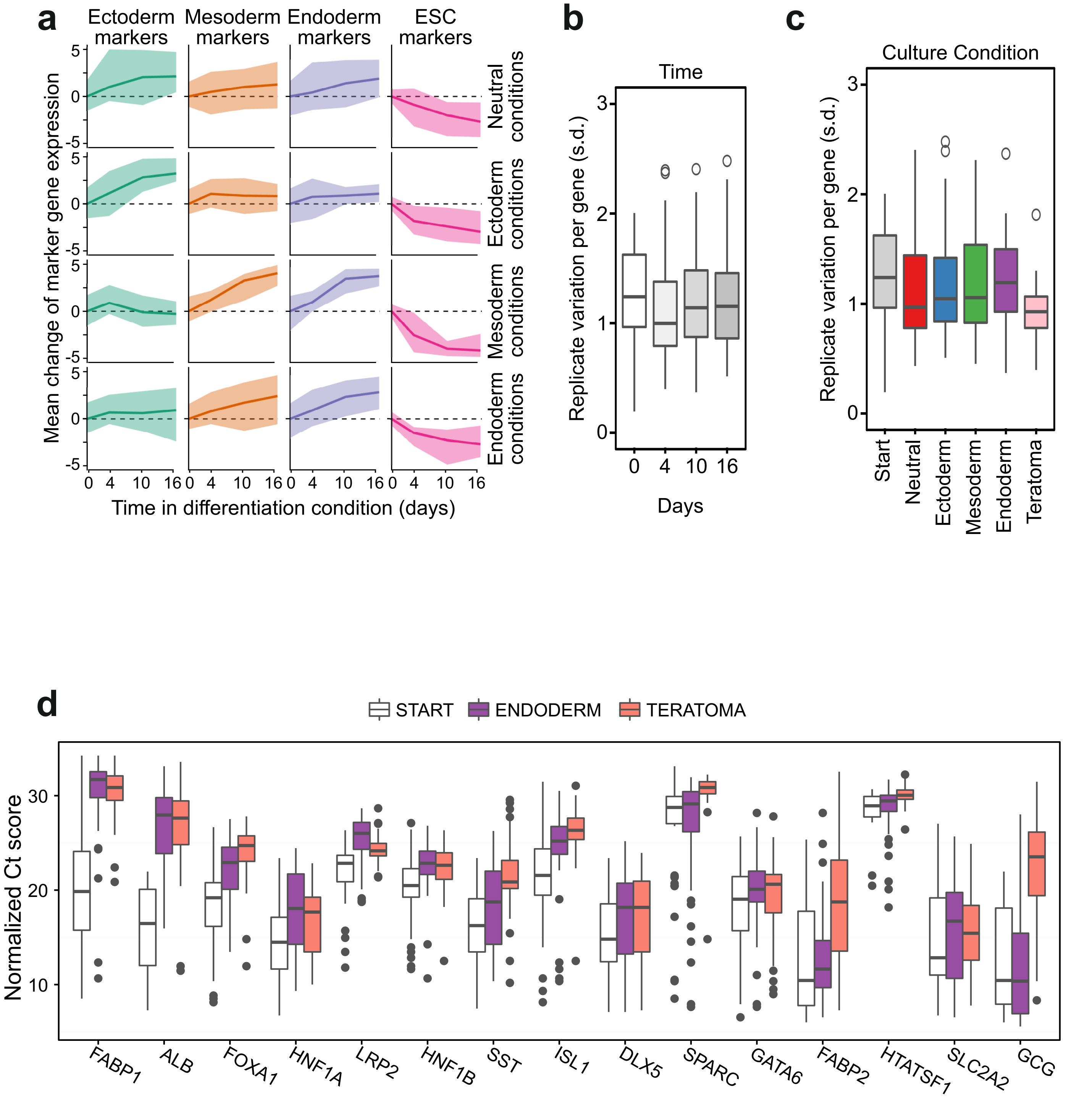

Supplementary Figure 2: Dynamics of embryoid body differentiation. a: Line plots showing the mean change (relative to day 0; log$_2$) of marker gene expression (Supplementary Table S3) as a function of time and averaged over all cell lines. Different sets of markers genes are shown from left to right: ectoderm markers, mesoderm markers, endoderm markers and markers of undifferentiated cells. Different cell culture conditions are displayed from top to bottom: neutral conditions, and conditions favoring ectoderm, mesoderm, or endoderm differentiation. Shaded contours indicate the minimum and maximum observed expression. **b:** Boxplots summarizing the average gene-wise standard deviation (s.d.) between replicate measurements at different time points of EB differentiation (across all culture conditions). Each data point corresponds to one standard deviation value calculated between all gene measurements for a pair of replicates. Replicates are those datasets with the same culture condition, time in culture, and from the same cell line. Boxes represent the interquartile range (IQR), the line is the median, whiskers extend to 1.5×IQR, and outliers are indicated as dots. **c:** Same as in panel b, but split per culture condition. All time points were considered. **d:** Boxplots of normalized expression scores for key endoderm genes in the starting condition at day 0 (white), after 16 days in endoderm differentiation conditions (purple), and in teratoma samples (pink). Each data point is one measurement for the given gene in one replicate. Data from all cell lines is considered. Boxes represent the interquartile range (IQR), the line is the median, whiskers extend to 1.5×IQR, and outliers are indicated as dots.

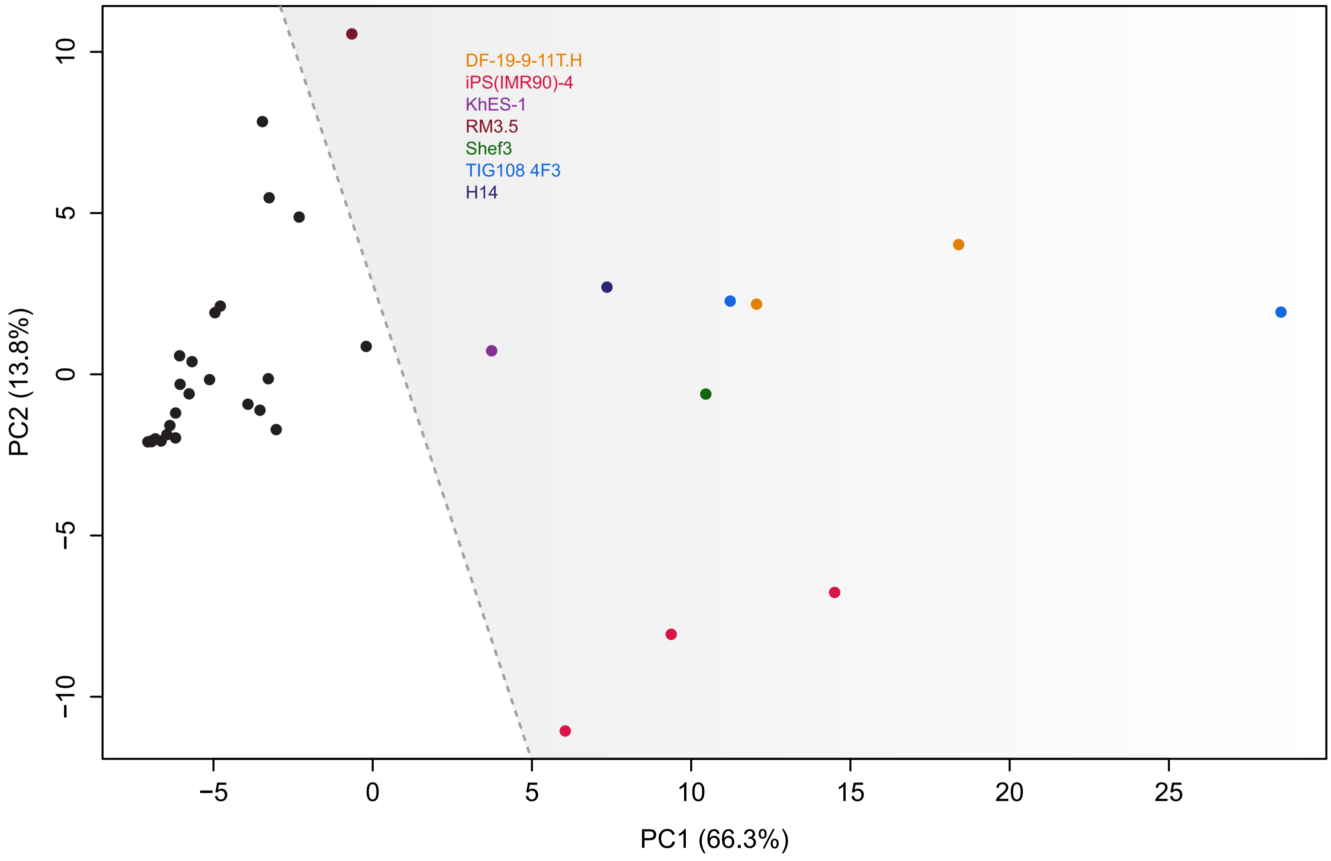

SUPPLEMENTARY FIGURE 3:Principal component analysis of undifferentiated PSC / yolk sac cell marker expression. Samples to the right of the dashed line, highlighted in colors show high expression of the markers of undifferentiated and yolk sac cells.

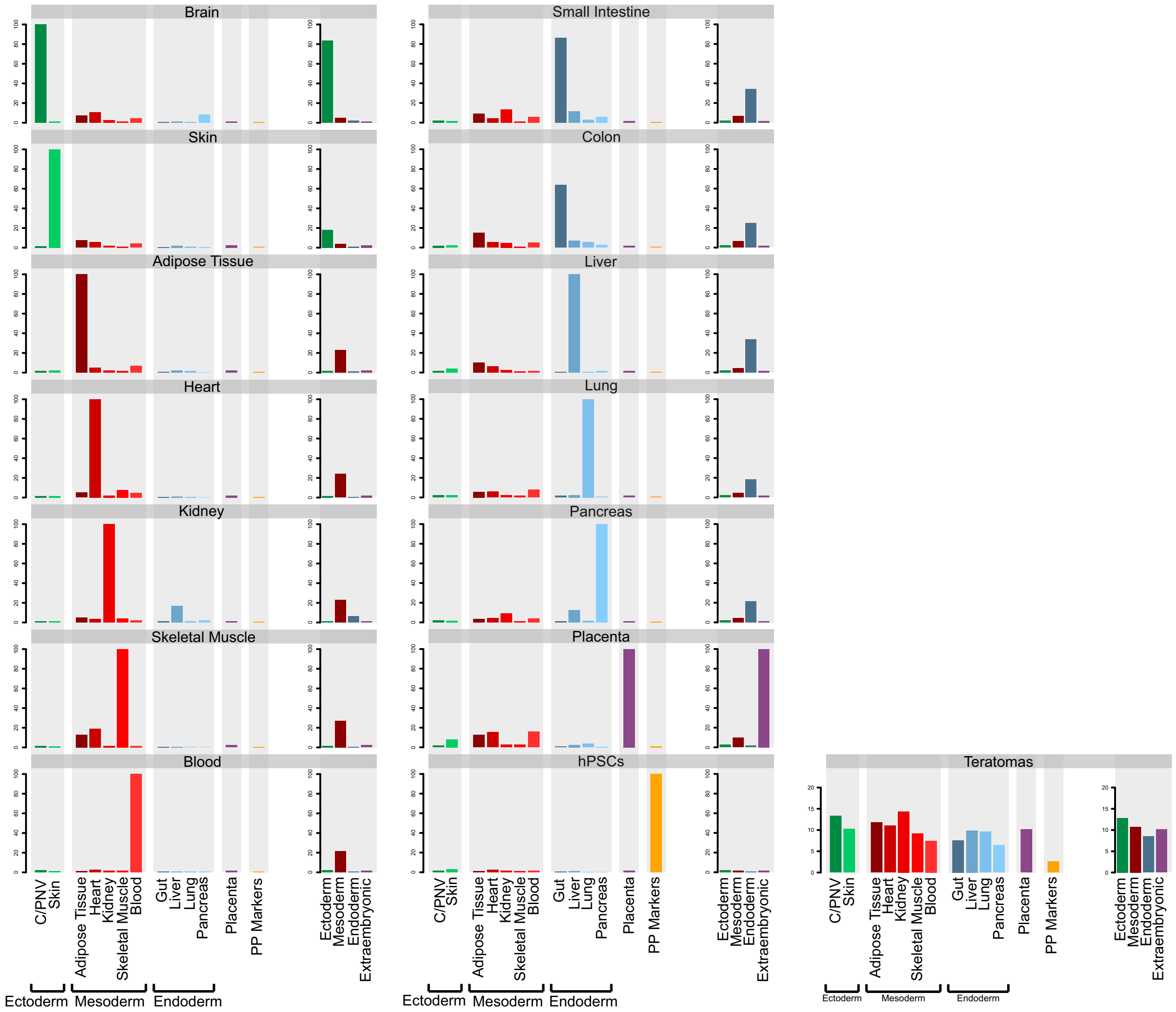

Supplementary Figure 4: TeratoScore grades of normal human tissues and of teratomas. Panels show expression values of genes representing somatic tissues from the three embryonic germ layers in a selection of respective reference tissues, human PSC lines and teratomas (Supplementary Table 4). Each tissue is represented by a subset within 100 tissue specific genes comprising the TeratoScore (Supplementary Table 3). The expression of each gene was calculated as the percentage of its expression in the tissue it represents, and the mean expression of each group of “tissue-specific” genes comprised the mean tissue expression. Lineage expression was calculated as the mean expression of all the tissues in that lineage. Note that each tissue showed a correct specific tissue- and lineage-expression, and that only teratomas showed an even expression of all lineage markers and expression of markers from all measured tissues.

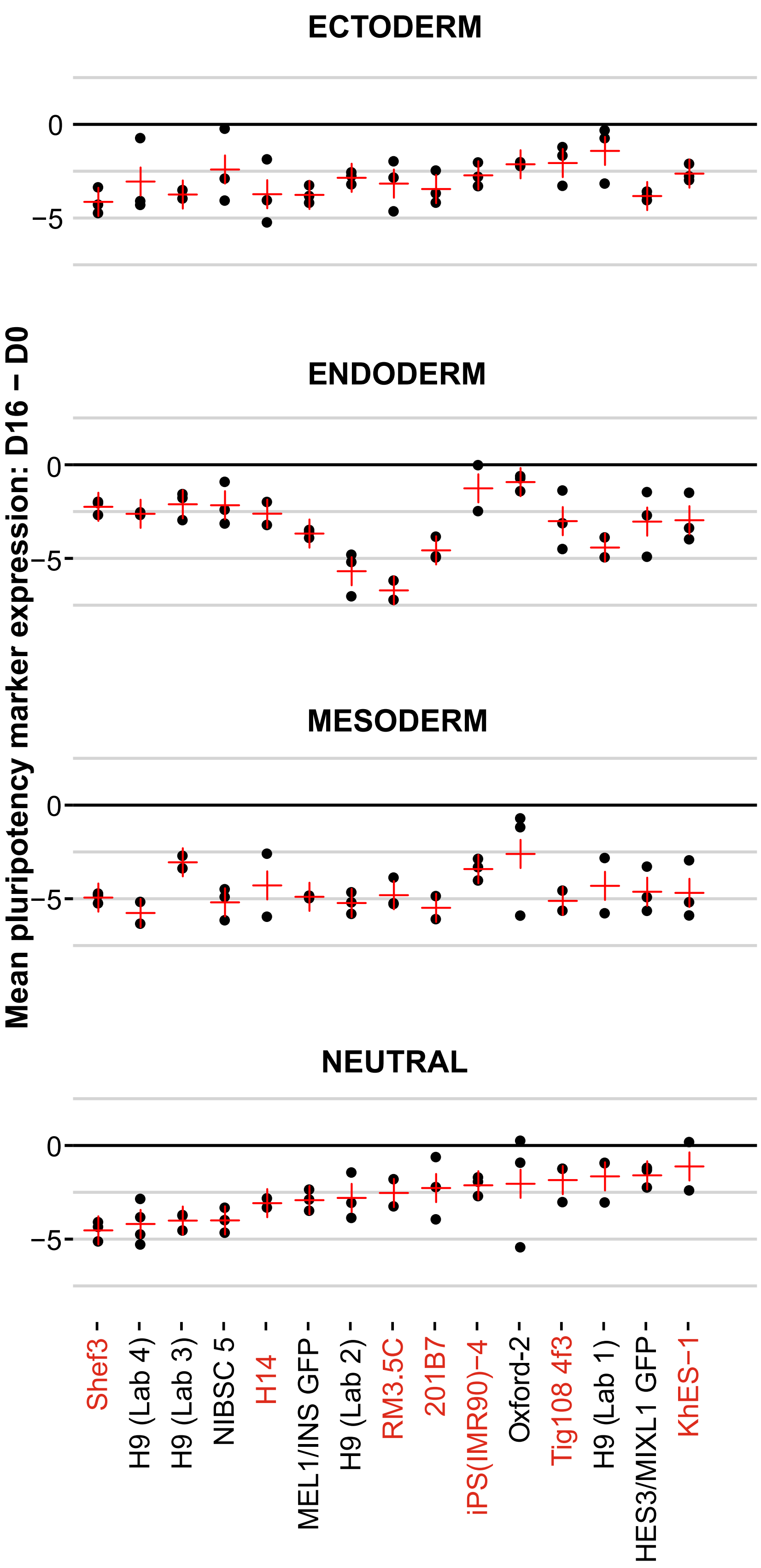

Supplementary Figure 5: **The persistence of undifferentiated stem cells in EBs at 16 days.** The dot plots summarize the average loss of expression (normalized Ct score, indicated on the y-axis) of 15 undifferentiated PSC marker genes (Supplementary Table 6) in replicates of EBs formed by each cell line after 16 days of culture under the different conditions, compared to starting conditions (mean across all replicates for the same cell line). Each dot corresponds to a single replicate dataset and the red crosses indicate the mean across all replicates. Cell lines have been sorted such that those with the greatest loss of PSC marker expression in neutral conditions are on the left and those with the highest residual expression are on the right. The cell lines highlighted in red had been found to produce xenograft tumors containing ECL and/or yolk sac elements as determined by histology or RNA-seq analysis (Table 1)
