# Supplementary material for: Assessment of established techniques to determine developmental and malignant potential of human pluripotent stem cells
Source: Nat Commun. 2018 May 15;9:1925. doi: 10.1038/s41467-018-04011-3 (PMC5954055; doi:10.1038/s41467-018-04011-3)
Supplement: Supplementary file 3 — Description of Additional Supplementary Files [file 41467_2018_4011_MOESM3_ESM.docx]

**Description of Additional Supplementary Files**

**File name: Supplementary Data 1**

**Description: Cell line growth and Differentiation conditions for embryoid body and Teratoma assays.** Complete table describing the culture conditions used in the embryoid body and teratoma assays.

**File name: Supplementary Data 2**

**Non-normalized Expression data for embryoid body differentiation assays.** Non-normalized gene expression data generated using the Fluidigm Biomark HD platform from the EB differentiation assays.

**File name: Supplementary Data 3**

**Description: Normalized Expression data for embryoid body differentiation assays.** Assay details and normalized gene expression gene expression data generated using the Fluidigm Biomark HD platform from the EB differentiation assays.

**File name: Supplementary Data 4**

**Description: List of genes comprising the TeratoScore analysis.** Genes specific to tissues originating from the three embryonic germ layers, the placenta and undifferentiated/primitive cells are listed. Genes were chosen by comparing RNA-seq expression data of 14 human body tissues, and identifying genes with high tissue-specificity (expressing over 8-fold higher in a given tissue, compared to the mean of all other tissues). The expression of these genes was further validated to be enriched in differentiated cells (see Methods). The ectoderm is represented by genes specific to the central and peripheral nervous system and skin; the mesoderm is represented by genes specific to adipose tissue, heart, kidney, muscle and blood cells; the endoderm is represented by genes specific to gut, liver, lung and pancreas cells.

**File name: Supplementary Data 5**

**Description: Gene-expression datasets used to adapt the TeratoScore algorithm to RNA-seq analysis.** Data sets from three different sources were used to adapt the TeratoScore algorithm to RNA-seq: *The Genotype-Tissue Expression project* (GTEx, [http://www.gtexportal.org](http://www.gtexportal.org/), Consortium GT. Human genomics. The Genotype-Tissue Expression (GTEx) pilot analysis: multitissue gene regulation in humans. *Science* **348**, 648-660 (2015), the NIH's Sequence Read Archive (SRA, <http://www.ncbi.nlm.nih.gov/sra)>and the current ISCI teratomas. For each sample the tissue of which it represent, its TeratoScore tissue classification and database of origin are shown. For GTEx samples, the RNA integrity number (RIN) and total ischemic time until RNA extraction are also provided.

**File name: Supplementary Data 6**

**Description: Plate 2148: QPCR assay designs for genes used in EB analysis.** Fluidigm Plate 2148 layout and assay design for the QPCR assays used in the EB analysis.

**File name: Supplementary Data 7**

**Description: Plate 2165: QPCR assay designs for genes used in EB analysis.** Fluidigm Plate 2165 layout and assay design for the QPCR assays used in the EB analysis.

**File name: Supplementary Data 8**

**Description: Metadata for the teratoma samples analysed by RNA-seq.**

**File name: Supplementary Data 9**

**Description: RNA-seq data for the teratoma analysis**.
